# Dataset about information technology governance: A survey in Colombian enterprises

**DOI:** 10.1016/j.dib.2023.109480

**Published:** 2023-08-10

**Authors:** Gina Maestre-Góngora, Diana Aponte

**Affiliations:** a Universidad Cooperativa de Colombia, Medellín, Colombia; b Universidad Cooperativa de Colombia, Bucaramanga, Colombia

**Keywords:** Information technology governance, Information technology project, Emerging technologies, Survey

## Abstract

This survey aimed to obtain and analyze relevant information on the implementation of best practices in information technology governance in Colombian organizations to identify current trends in Information Technology (IT) project management, the impact of IT governance, and the use of emerging technologies. A semistructured survey was conducted among the IT professionals of Colombian companies of different sizes and economic sectors between 2019 and 2022. The survey was designed considering international references, such as ISACA, and following the Kimball methodology to guide the analysis. A total of 252 responses were collected, and 237 records were analyzed. It was concluded that the successful implementation of IT governance can improve efficiency, productivity, decision-making, information security, competitiveness, and customer service quality. However, the Small and Medium-sized Enterprises (SME's) face challenges such as a lack of skilled human resources, resistance to change, and high implementation costs. To address these challenges, strategies such as prioritizing investments, focusing on internal talent, communicating benefits and expected results, and investing in the training of the organization's personnel are suggested.

Specification Table

**Table:** 

Subject	Business, management, and decision sciences/management of technology and innovation
Specific subject area	Information technology governance
Type of data	Table
How the data were acquired	The data were obtained from a semistructured online survey based on the ISACA Governance Enterprise Information Technology Survey [1]
Data format	Raw
Description of data collection	The data were collected from IT staff in Colombian companies of different sizes and economic sectors using Google Forms from 2019 to 2022. The original survey was conducted in Spanish and translated into English. The raw Spanish data can be verified in [2]. Data were obtained from a semistructured online survey based on the ISACA Governance Enterprise Information Technology Survey [1]. The objective of this study was to identify respondents’ perceptions of practices related to technology governance in their organizations. A total of 252 responses were collected over a period of 4 years, and after preprocessing and data cleaning, 232 records were selected for analysis. Convenience sampling was performed according to ease of access and availability of sample respondents . Two criteria were used to select the sample respondents. First, the respondent must be a student of postgraduate studies related to information technology management, that is, they must have a basic interest in and knowledge of ICT. Second, they work in an organization and can recognize the role of IT in the organization. From the data, cross-sectional analyses can be conducted to evaluate how employees from different roles and positions perceive the strategic alignment of IT, projections and incidence of IT projects, and emerging technologies in organizations
Data source location	Online survey: https://forms.gle/RZ4Be1pUJqvHmCHNA Country: Colombia
Data accessibility	Repository name: Zenodo Dataset, survey, and Codebook: https://zenodo.org/record/8077693
Related research article	Trujillo-Lambert, Y., Osorio Sanabria, M., Maestre-Góngora, G., & Astudillo, H. (2023). Information Technology Governance in Colombian Small and Medium Companies: An Exploratory Study Using Data Analysis. AiBi Revista De Investigación, Administración E Ingeniería, 11(1), 66–74. DOI:https://doi.org/10.15649/2346030X.3083

## 1 Value of the Data

• The data can be used in academia to identify challenges and trends in IT governance practices in organizations of different sizes or economic sectors. In this sense, students and professors in the field of information technology management can have a source of data from practice for developing research agendas on issues related to strategic alignment of IT in the organization, benefits and problems in IT projects, and emerging IT use.
• The data can be useful to organizational leaders, decision-makers, and IT service managers because it shows the opportunities and constraints that organizations have in managing information technology, enabling them to establish strategies for best practices of IT governance to increase the impact and generate added value from IT investments.
• The data can be used to replicate this study in other regions or several contexts simultaneously for comparative analysis of the results. This  study can also be conducted with a focus on a specific target audience (e.g., only public, private, or specific-size organizations). The questions can be expanded or adapted and the response scales can be adjusted (Appendix A).
• The data can be used to conduct cross-sectional analyses, including descriptive, diagnostic, or predictive models, to identify and correlate variables regarding the impact of different initiatives and actions on the state of IT governance in organizations. For example, variables such as IT benefits, reasons for project cancelation, technologies used, and level of management involvement could be analyzed, taking into account the size of the organization or economic sector or according to the position of the respondent.
• The data are available in English and Spanish, which can facilitate their use in different contexts, mainly Latin America, where these studies are limited.

## 2 Objective

The objective of this study is to analyze and understand the importance, status, and trends of IT governance in Colombian organizations, considering variables such as the characterization of organizations, strategic alignment of IT, projections, and incidence of IT projects, in addition to the use of emerging technologies such as big data, artificial intelligence, virtual/augmented reality, and robotics, presenting a broader perspective that allows the identification of opportunities for adoption and use of IT.

## 3 Data Description

Title dataset: Survey information technology governance in Colombian enterprises

Variables: Qualitative and quantitative variables

Format: CSV

Online survey: Google Forms

Country: Colombia

City/Region: Location where the survey was completed

**Table:** 

• Bogotá • Medellín • Barranquilla • Bucaramanga • Valle del Cauca • Huila • Villavicencio • Cartagena • Cundinamarca • Quibdó	• Antioquia • Casanare • San Andrés Islas • Montería • Santa Marta • Ibagué • Córdoba • Pasto • Boyacá • Cauca	• Neiva • Cúcuta • Cesar • Sincelejo • Quindío • Valledupar • Riohacha • Yopal • Pereira • Manizales

Records collected: 252

Records analyzed: 237

Release date: 02–10–2019

Closing date: 09–30–2022

The survey was applied mainly to IT professionals who are also students of a graduate program in information technology at three universities in Colombia (Master of Information Technology Management 2019–2022, Master of Information Management Universidad de Medellín 2019–2020, and Master of Information Technology Governance Universidad del Norte 2022).

Considering that it is a survey directed to a specific target audience (master's students in IT), the objective of the survey was presented to the respondents synchronously. Moreover, an invitation was sent to the students at the beginning of the academic semester. The link is then shared through academic management platforms such as Brightspace and Moodle, where it remains open to be answered optionally.

**Fig. 1:**
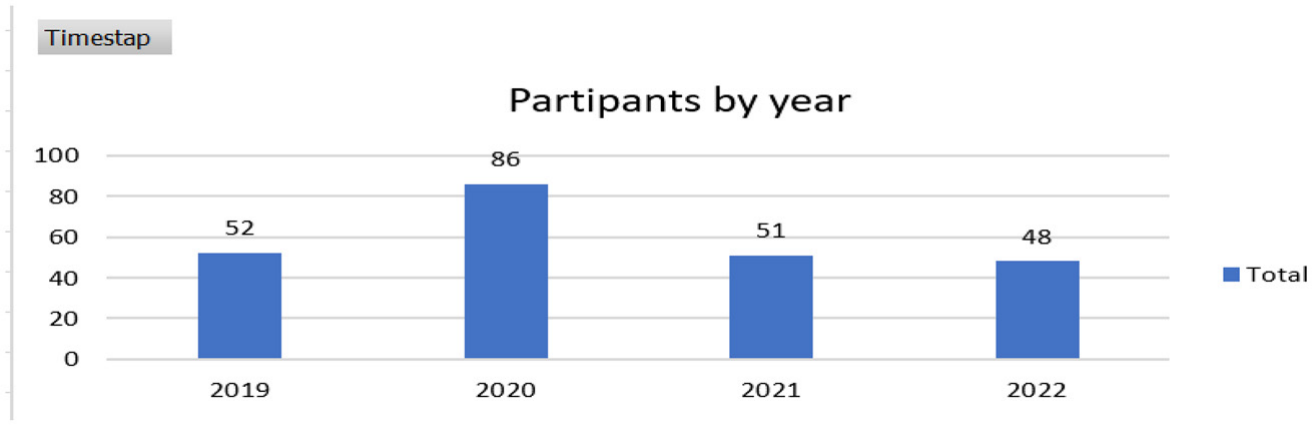
Number of participants by year.

The data were sent and collected in four rounds corresponding to the survey application years (Y1_2019, *n*=  52; Y2_2020, *n*=  86; Y3_2021, *n*=  51; and Y4_2022, *n*=  48), as shown in Fig. 1. The response rate was higher than 90% considering that it was conducted with a defined focus group, where the respondents were directly motivated, and the preliminary results were discussed from the academic exercise.

The data codebook, available as a complementary file in the Zenodo repository, contains a description of the column names, as well as the applied survey with its question and answer options [3].

**Fig. 2:**
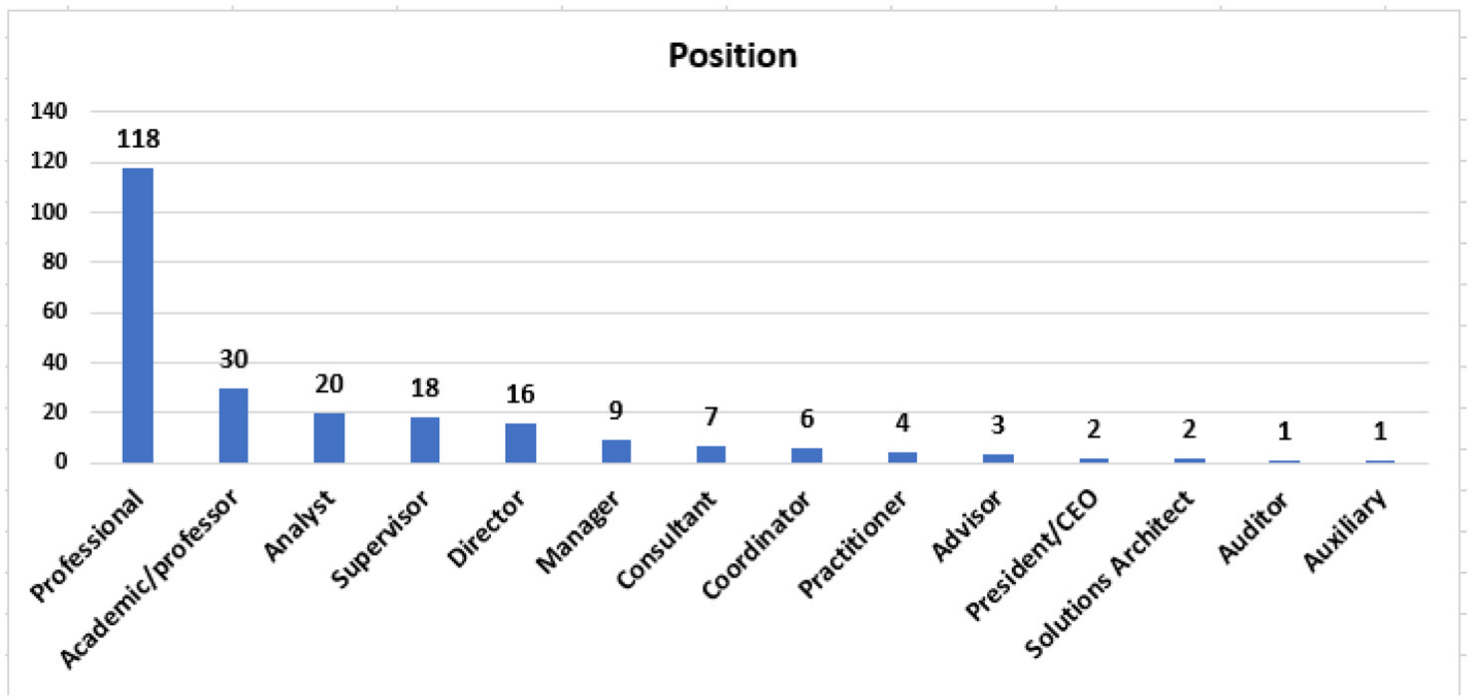
Positions closest to your level of work.

**Fig. 3:**
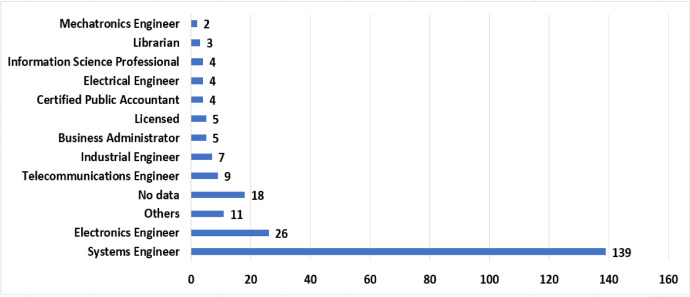
Undergraduate degree of participants.

The demographic information of the participants and their organizations are presented in Fig. 2 (position), Table 1 (specific position), Fig. 3 (undergraduate degree) and Table 2 (information of organizations).

**Table 1:** Specific position in the organization.

Position_Specific	%
Software engineer/Software developer	22,4
Analyst_Advisor	12,7
Academic	11,8
IT_Leader/IT administrator	10,0
Professional	9,3
Coordinator	7,2
Manager	5,5
Director	4,2
Instructor	4,2
Auxiliary_Assistant	3,0
Consultant	2,1
Contractor	2,1
Technician	1,3
Accountant	0,8
FTTH project manager	0,4
Geologist	0,4
Metrologist	0,4
Municipal project bank formulator and administrator	0,4
Process intern	0,4
Senior QA automation engineer	0,4
Supernumerary	0,4
Technology inspector	0,4

**Table 2:** Demographic information of organizations.

Options	Size_Organization	Options	Sector_Organization
0–10	13	Mixed	20
10–50	26	Private	134
50–250	35	Public	83
Over 250	163	**Total**	237
Total	237		

Options	Sector_Organization	Options	Coverage Organization

Education	73	International	58
Goods and services	10	Local/Regional	62
Government	41	National	117
Health	15	**Total**	237
Industrial/Commercial	39		
IT services	59		
**Total**	**237**		

**Fig. 4:**
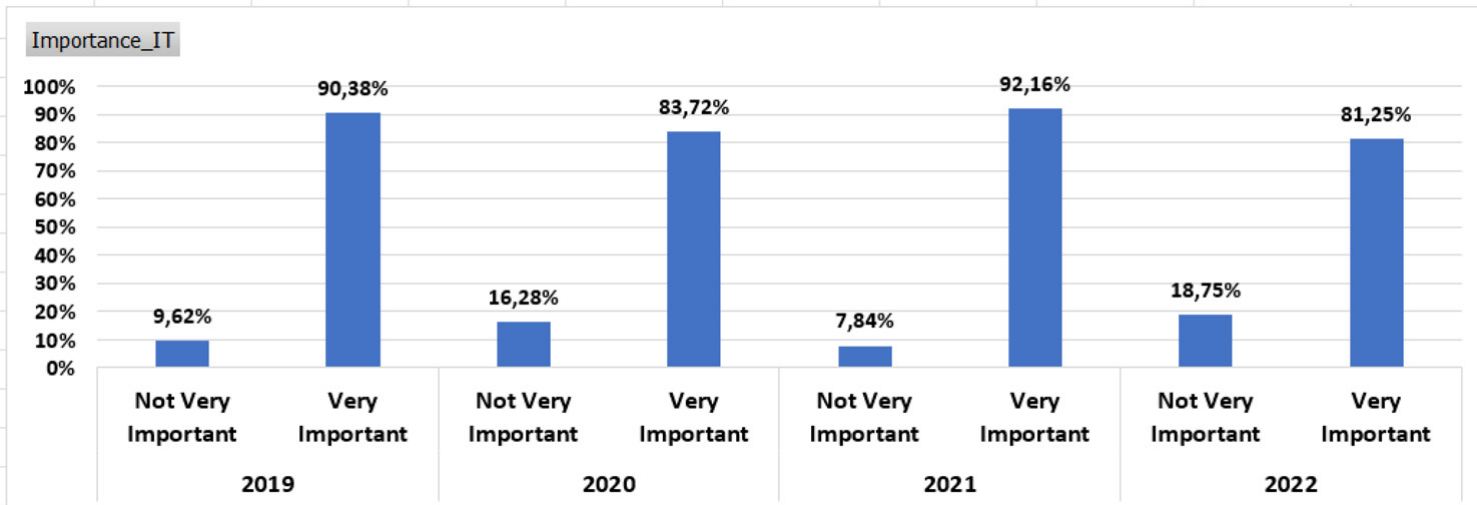
Importance of IT by year.

**Fig. 5:**
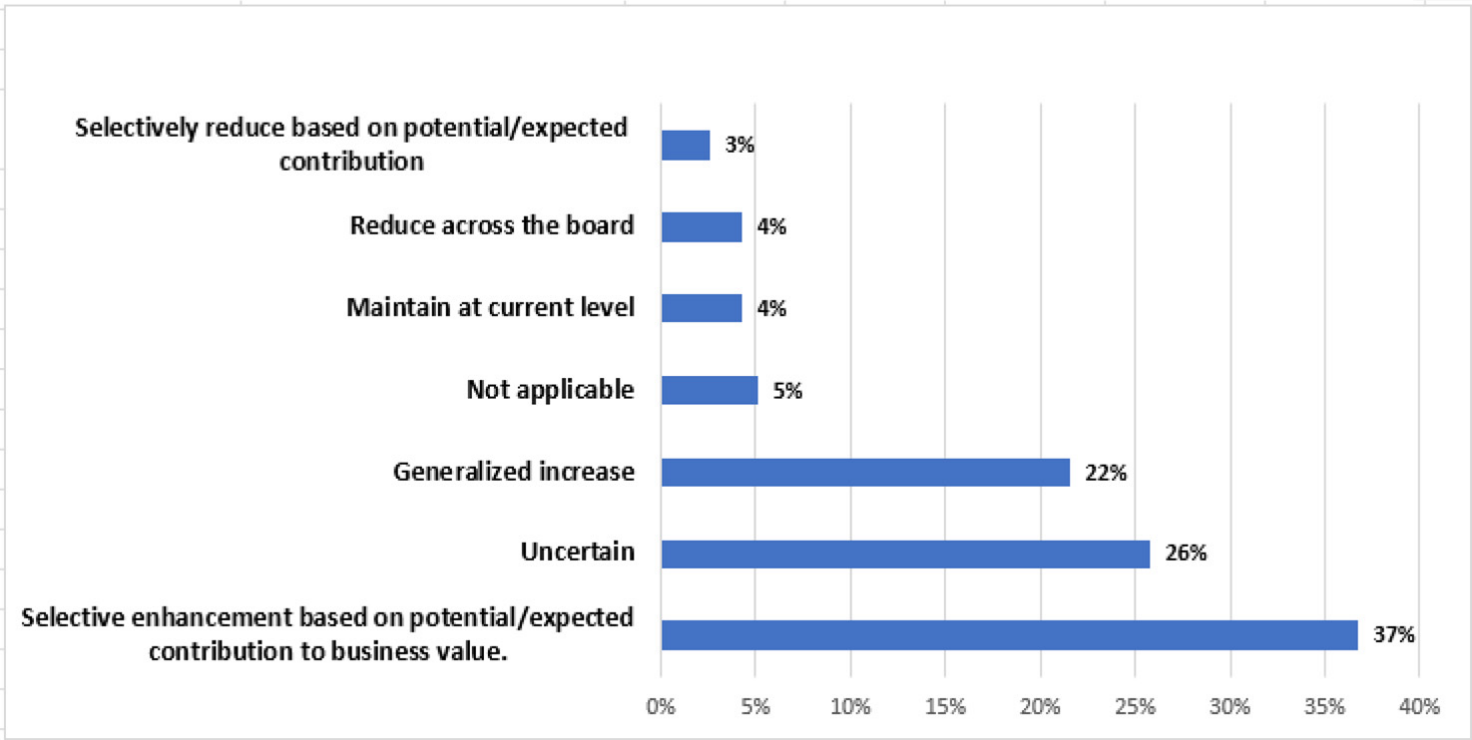
IT investment plan.

**Fig. 6:**
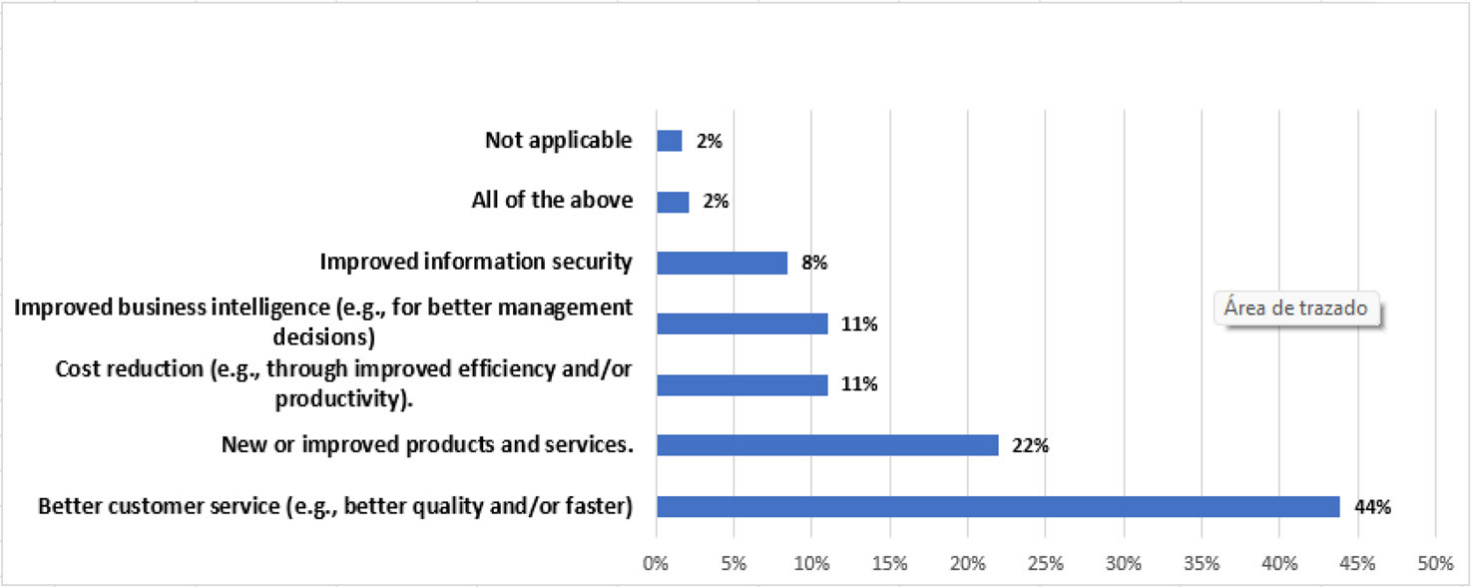
Benefits of IT investments.

Fig. 4, Fig. 5, Fig. 6

The dataset descriptions are shown in Tables 3 and 4.

**Table 3:** Dataset description.

ID_Register	Type	Question type
1_Timestap	Date and Time [DD.MM.YYYY HH: mm]	Metadata
2_Position_Nearby	String (defined items)	Single-choice options
2_1_Position_Specific	String	Open answer
2_2_Professional_Title	String	Open answer
3_Number_of_Employees_Org	String (defined items)	Single-choice options
4_Coverage_Org	String (defined items)	Single-choice options
5_City_Org	String	Text input
6_Country_Org	String	Text input
7_Type_Org	String (defined items)	Single-choice options
8_Sector_Org	String (defined items)	Single-choice options
9_IT_Oficce	String/Boolean	Single-choice options
10_Importance_IT	String (defined items)	Single-choice options
11_IT_involvement_level	String (defined items)	Single-choice options
12_IT_investment_plan_Current	String (defined items)	Single-choice options + others
13_Current use_IT	String (defined items)	Multiple-choice options + others + none
14_Future use_IT	String (defined items)	Multiple-choice options + others + none
15_Problems_IT	String (defined items)	Multiple-choice options + others + none
16_Use_marco/estándar_TI	String (defined items)	Single-choice options
17_TOGAF	String/Boolean	Single-choice options
17_ITIL	String/Boolean	Single-choice options
17_ZACHMAN	String/Boolean	Single-choice options
17_COBIT	String/Boolean	Single-choice options
17_ISO	String/Boolean	Single-choice options
17_CMMI	String/Boolean	Single-choice options
17_OTHERS	String/Boolean	Single-choice options
18_Problems_IT_Last6Month	String (defined items)	Multiple-choice options + others + none
19_Cancel_project_IT	String (defined items)	Single-choice options
20_If CP_select the reason	String (defined items)	Single-choice options + others + none
21_Benefits	String (defined items)

**Table 4:** Description of dataset fields.

ID_Register	Description	Options
1_Timestap	Date the survey was conducted	Metadata
2_Position_Nearby	The position is closest to your level of work	Analyst Professional Supervisor Manager Director Vice president President/CEO External consultant Academic Intern
2_2_Professional_Title	Education (undergraduate degree)	Open answer
2_1_Position_Specific	Specific position in the organization	Open answer
3_Number_of_Employees_Org	Number of people employed in the company, including all branches, divisions, and subsidiaries	0–10 10–50 50–250 Over 250
4_Coverage_Org	Territorial scope where the company is present	International Local/Regional National
5_City_Org	The location where the person completing the survey works	Open answer
6_Country_Org	Country where the person completing the survey works	Open answer
7_Type_Org	Origin of the capital of the organization or company where the person completing the survey works	Private Public Mixed
8_Sector_Org	The economic sector of the organization or company where the person completing the survey works	Industrial/Commercial Technology services Government Education Goods and services Health
9_IT_Oficce	Existence of an IT department or management in the organization	Yes No
10_ Importance_IT	Importance of information and technology for the strategy and vision of your company for the executive team	Very important Not very important Do not know
11_IT_involvement_level	Level of involvement of the company's management in IT governance	High Low Moderate Uncertain
12_IT_investment_plan_Current	IT-related investment plan for the next 12 months	Selective growth based on potential/expected contribution to business value Uncertain Generalized increase Reduce across the board Freeze at the current level Reduce selectively based on potential/expected contribution to business value Do not know
13_Current use_IT	Current use of emerging and new technology to support processes	Data mining Business intelligence Big data Artificial intelligence Virtual reality/Augmented reality Social networking Robotics/Process automation Others None
14_Future use_IT	Technologies that the organization where the respondent works plans to implement in the short- or medium-term	Data mining Business intelligence Big data Artificial intelligence Virtual reality/Augmented reality Social networking Robotics/Process automation Others None
15_Problems_IT	Aspects that the company has experienced in the last 12 months as a result of an IT-related problem/incident	Incurring unexpected expenses Reputation was damaged Customer satisfaction was reduced Cost reduction opportunities were delayed or lost A competitor beat my company to market None Other
16_Use_marco/estándar_TI?	Does your company use a framework/standard for governance and management/governance/EA or IT services?	Yes No I do not know
17_TOGAF	If yes, which framework/standard is used? TOGAF	Yes = 1 No = 0
17_ ITIL	Does your organization use a framework/standard for governance and management/governance/EA or IT services? ITIL	Yes = 1 No = 0
17_ ZACHMAN	If yes, which framework/standard is used? ZACHMAN	Yes = 1 No = 0
17_ COBIT	Does your company use a framework/standard for governance and management/governance/EA or IT services? COBIT	Yes = 1 No = 0
17_ISO	If yes, which framework/standard is used? ISO	Yes = 1 No = 0
17_CMMI	Does your company use a framework/standard for governance and management/governance/EA or IT services? CMMI	Yes = 1 No = 0
17_ OTHERS	If yes, which framework/standard is used? OTHERS	Yes = 1 No = 0
18_Problems_IT_Last6Month	IT-related problems experienced by the company in the last 12 months	High IT cost with low or unknown return on investment Project cost overruns Security breaches Inadequate IT staffing Inadequate disaster recovery or business continuity measures Lack of innovation Serious IT operation incidents Outsourcing issue None Other Do not know
19_Cancelado_proyecto_TI	Termination or cancelation of an IT-related project before it is fully implemented	Yes No Do not know
20_ If CP_select the reason	If yes to the above answer, please select the reason	Changes in business needs Failed to deliver what waspromised Exceeded budget Did not support business strategy No other reason for cancelation Other reasons for cancelation
20-benefits	Perceived benefits of IT adoption or implementation	Improved customer service New or improved products and services Cost reduction Improved business intelligence Improved information security All of the above Not applicable

## 4 Experimental Design, Materials and Methods

The survey design was based on the research findings reported by ISACA's IT Governance Institute [1]. Initially, three questions were proposed as instrument design guidelines (1) What is the perception, recognition, and acceptance of IT governance in Colombian organizations? (2) Do Colombian companies’ sizes and origins have any connotations regarding failures in technological projects? (3) What have been the best practices in IT adoption in recent years? The survey was conducted in Spanish because it was developed in a Hispanic country and used an online form; and the original data in Spanish are available in [2]. Once the data were preprocessed, a new dataset was generated in English, which is described in Tables 1 and 2 and available in [4].

The study design applies the design principles of Kimball's methodology [5].

Focusing on the business: We focused on the value generated and the requirements of the project, managing to interpose specific relationships that would allow the fulfillment of the research objectives (survey), designed to answer three specific questions.

Develop an adequate information infrastructure: The infrastructure that would generate complete, specific, and easy-to-use information was determined, with tools available for developing the project. Thus, an adequate data model was defined and designed for a project that included the necessary dimensions and facts to answer the analysis questions.

Deliver in significant increments: A plan of incremental deliveries was defined making three deliveries and providing value to the project by allowing the delivery of the solution in short periods; seeking accessibility and better visualization; and focusing on the research questions. Design and construction tools for reports, graphs, and control panels were used to analyze and visualize the data.

The survey consisted of an introduction, informed consent form, and questions that were grouped as follows:

• Ten demographic questions were asked about the respondents and their organizations.
• Five questions were about the current implementation of governance enterprise Information Technology and the strategic alignment of IT in the organization, the role of IT within the organization, benefits, and investments.
• Ten questions were related to IT projections, problems in IT projects, and use of emerging technologies.
• A closing question that the respondent answered freely allowed us to know their reflections about IT.

A full reference of the survey questions and predefined multiple-choice options is provided in Appendix A [3].

From this original dataset, data transformation was performed to make the data more useful, including the following steps:

• Translation of the Spanish dataset to English
• Identification and correction of missing data
• Correction of erroneous or inconsistent data
• Categorization of open-ended responses to facilitate analysis
• Classification of the data as necessary to focus on a specific group of respondents or a specific question, such as the level of business management
• Separation of multiple responses into separate fields for multiple-choice questions, creating new Boolean columns for analysis
• Selection of columns to eliminate prioritizing those that allow the anonymization of respondents, open-ended questions, and questions that are contextual information or irrelevant to this publication

Once the data were extracted and transformed, descriptive data analysis was initiated, including creating graphs and tables to summarize the data, identifying patterns and trends, and performing more advanced statistical analysis.

The profile of the respondents was identified, along with general information about the companies in which they worked. Visualization tools, such as histograms, stacked bars, pie charts, and ring graphs, were used to help interpret the information and improve its analysis, making the following deliverables. The first installment aimed to identify the key findings of the survey for each of the questions asked, allowing the identification of patterns and an interactive and easy-to-read data presentation. The second installment sought to have more detail among the variables, relate the size of the organization with other questions, focus on the answers by candidate profile, add a timeline to the results, and add sheets for better visualization. The third installment focuses on three design questions that allow the identification of the perception of IT in organizations and their governance.

The results of this study are as follows:

• Of the respondents, 84.8% belonged to tactical positions in the organization, followed by 11.3% and 3.8% of strategic and operational profiles, respectively.
• In 86% of the companies, there is an IT department and IT involvement, and importance levels are high. In the remaining 6% of the cases, despite having an IT department with a high level of involvement, these are not considered very important.
• Of the companies with an IT department, 45% use an IT framework or standard, and in this group, they may use several frameworks.
• In large and multinational companies, the biggest impact of IT problems or incidents is unexpected expenses. Likewise, problems that could arise are focused on cyberattacks; therefore, one of the main problems is insufficient IT personnel for this type of failure.
• In SMEs, the biggest impact of IT problems or incidents is to incur unexpected expenses. Likewise, problems that could occur are unintentional errors by employees, with insufficient IT personnel being the primary problem.
• The main reason for project cancellations is changes in business needs, regardless of company size.
• In the last four years the main benefits of IT implementations, the investment plan, and the use of emerging technologies are focused on improving customer service; the collective increase based on the potential/expected contribution to business value; and data mining/business intelligence/big data, and social networks, respectively.
• For companies, especially large ones, there is a clear understanding that IT governance is essential for ensuring regulatory compliance, information security, and business continuity. In these companies, the top management is involved in defining and overseeing IT governance policies. In smaller companies, there may still be a lack of understanding of the importance of IT governance, which may be perceived as an additional cost or administrative burden, with IT governance being less structured and focused on technical solutions rather than IT processes.

The survey design can be used to replicate the study targeting specific audiences (IT department staff, management and strategic staff of organizations, or technology users) in one or several organizations. Additionally, the study can be targeted at organizations of a specific size or economic sector using cross-sectional or longitudinal approaches. The study can be conducted using probability sampling to perform qualitative or quantitative analysis of all or some variables of interest.

## Ethics Statements

It was not mandatory to include the endorsement of the ethics committee for the surveys of this project at the Universidad Cooperativa de Colombia in 2019. The guidelines of Colombian public policy were followed, which were established to guarantee and respect the privacy of personal data on which the authors perform treatment and to fully comply with the parameters established in Article 15 of the political constitution of Colombia, law 1587 of 2012, regulatory decrees 1377 of 2013 and 886 of 2014 today incorporated in chapters 25 and 26 of single decree 1074 of 2015 and the other rules that regulate it.

The data anonymization process was conducted by eliminating from the raw data the identification (first and last names) of the respondents and the name of the organization for data analysis. It should be noted that identification data in the survey were not mandatory.

## CRediT authorship contribution statement

**Gina Maestre-Góngora:** Conceptualization, Methodology, Validation, Formal analysis, Writing – review & editing, Project administration, Funding acquisition. **Diana Aponte:** Conceptualization, Formal analysis, Data curation, Validation.

## Data Availability

Survey : Information Technology Governance in Colombian Enterprises (Original data) (Zenodo).

## Appendix A

The transcripts and translations of the full questionnaire are as follows.
